# Correction: Villalba et al. Development and Validation of Three Triplex Real-Time RT-PCR Assays for Typing African Horse Sickness Virus: Utility for Disease Control and Other Laboratory Applications. *Viruses* 2024, *16*, 470

**DOI:** 10.3390/v18090963

**Published:** 2026-09-02

**Authors:** Rubén Villalba, Cristina Tena-Tomás, María José Ruano, Marta Valero-Lorenzo, Ana López-Herranz, Cristina Cano-Gómez, Montserrat Agüero

**Affiliations:** 1 Laboratorio Central de Veterinaria, Ministry of Agriculture, Fisheries and Food, 28110 Algete, Spain; rvillalba@mapa.es (R.V.); mruanor@mapa.es (M.J.R.); mvalero@mapa.es (M.V.-L.); alherranz@mapa.es (A.L.-H.); ccano@mapa.es (C.C.-G.); 2 Tecnologías y Servicios Agrarios, S.A. (TRAGSATEC), 28037 Madrid, Spain; at_algete9@mapa.es

## Error in Table 1

In the original publication [1], there was a mistake in Table 1. Serotype-specific rRT-PCR primers and probes targeting VP2 AHSV genes for serotypes 1–9. F: Forward, R: Reverse, P: Probe. as published. The mistake lies in the nucleotide sequences of some primers and/or probes, specifically Serotype 2 (AHS-2F), Serotype 3 (AHS-3F and AHS-3R), Serotype 7 (AHS-7F, AHS-7R, and AHS-7P), and Serotype 9 (AHS-9R and AHS-9P).

**Table 1:** Serotype-specific rRT-PCR primers and probes targeting VP2 AHSV genes for serotypes 1–9. F: Forward, R: Reverse, P: Probe.

AHSV	Primers (F/R) and Probe	Sequence 5′-3′
Serotype 1	AHS-1F	GCAAGCGCTGGCACTTG
	AHS-1R	TTCGAACTCATTCCTTACATCAACA
	AHS1P	FAM-AATGTCTTAGATCGTCAACT-MGB
Serotype 2	AHS-2F	CGGAAACTYTGTATTGCCAAA
	AHS-2R	TTGTCRTCCTGATCAACCCTAA
	AHS-2P	Cy5-TGAAGGTGCTTACCCGATCTTTCCACA-BBQ
Serotype 3	AHS-3F	AATTATTACAGCGGAGAATGCAGTT
	AHS-3R	GGTTATGAGTGGGGTGCGA
	AHS-3P	FAM-AGAGTTGAGGTTGCGGGA-MGB
Serotype 4	AHS-4F	TGAGGTGGAACACGAYATGTC
	AHS-4R	GATATGCCCCCTCACAYCTGA
	AHS-4P	VIC-TATCGGRATTTATGTACAATGAG-MGB
Serotype 5	AHS-5F	GAAGAGACAGGCGATTCAAATGA
	AHS-5R	AAAGCCACCCTTTTTGGTACAAA
	AHS-5P	NED-TGTTGARATGCTGAGGC-MGB
Serotype 6	AHS-6F	AGCCAGGGCTTCTTTGCA
	AHS-6R	CTCATGTTCAACCCACTGTACATTAA
	AHS-6P	VIC-GTCATCACCGTAAGCG-MGB
Serotype 7	AHS-7F	AGCCAGGGCTTCTTTGCA
	AHS-7R	CTCATGTTCAACCCACTGTACATTAA
	AHS-7P	VIC-GTCATCACCGTAAGCG-MGB
Serotype 8	AHS-8F	GAAATTATCAGCGGACTGACTAAGAA
	AHS-8R	AAACATCTACCTTTTGCGAATCTTG
	AHS-8P	NED-ACGTGATTCTTTTCCC-MGB
Serotype 9	AHS-9F	TACTGTGTCGGTGAGGGATTTT
	AHS-9R	GCCACGACCGGATATGA
	AHS-9P	FAM-AAACAAACGAAATGTGAA-MGB

The corrected Table 1. Serotype-specific rRT-PCR primers and probes targeting VP2 AHSV genes for serotypes 1–9. F: Forward, R: Reverse, P: Probe. appears below. The authors state that the scientific conclusions are unaffected. This correction was approved by the Academic Editor. The original publication has also been updated.

**Table 1:** Serotype-specific rRT-PCR primers and probes targeting VP2 AHSV genes for serotypes 1–9. F: Forward, R: Reverse, P: Probe.

AHSV	Primers (F/R) and Probe	Sequence 5′-3′
Serotype 1	AHS-1F	GCAAGCGCTGGCACTTG
	AHS-1R	TTCGAACTCATTCCTTACATCAACA
	AHS1P	FAM-AATGTCTTAGATCGTCAACT-MGB
Serotype 2	AHS-2F	CGGAAACYATGTATTGCCAA
	AHS-2R	TTGTCRTCCTGATCAACCCTAA
	AHS-2P	Cy5-TGAAGGTGCTTACCCGATCTTTCCACA-BBQ
Serotype 3	AHS-3F	AATTATTACAGCGGARAATGCAGTT
	AHS-3R	TCGCACCCCACTCATAACC
	AHS-3P	FAM-AGAGTTGAGGTTGCGGGA-MGB
Serotype 4	AHS-4F	TGAGGTGGAACACGAYATGTC
	AHS-4R	GATATGCCCCCTCACAYCTGA
	AHS-4P	VIC-TATCGGRATTTATGTACAATGAG-MGB
Serotype 5	AHS-5F	GAAGAGACAGGCGATTCAAATGA
	AHS-5R	AAAGCCACCCTTTTTGGTACAAA
	AHS-5P	NED-TGTTGARATGCTGAGGC-MGB
Serotype 6	AHS-6F	AGCCAGGGCTTCTTTGCA
	AHS-6R	CTCATGTTCAACCCACTGTACATTAA
	AHS-6P	VIC-GTCATCACCGTAAGCG-MGB
Serotype 7	AHS-7F	GAGTGGGGCGCAACAAATC
	AHS-7R	CCGGATATGCACCCTCACAT
	AHS-7P	VIC-CGATATCTGAGTTCATGTATGAA-MGB
Serotype 8	AHS-8F	GAAATTATCAGCGGACTGACTAAGAA
	AHS-8R	AAACATCTACCTTTTGCGAATCTTG
	AHS-8P	NED-ACGTGATTCTTTTCCC-MGB
Serotype 9	AHS-9F	TACTGTGTCGGTGAGGGATTTT
	AHS-9R	GGCACGACCGGATATGA
	AHS-9P	FAM-TTCACATTTCGTTTGTTT-MGB
