# Supplementary material for: Development and Validation of Three Triplex Real-Time RT-PCR Assays for Typing African Horse Sickness Virus: Utility for Disease Control and Other Laboratory Applications
Source: Viruses. 2024 Mar 20;16(3):470. doi: 10.3390/v16030470 (PMC10974454; doi:10.3390/v16030470)
Supplement: Supplementary file 1 [file viruses-16-00470-s001.zip › viruses-2896832-supplementary.pdf]

viruses

MDPI

Table S1. Analysis of serial dilutions of the nine AHSV prototype strain (serotypes 1 to 9) by GS rRT-PCR method described by Agüero, 2008 and TS rRT-PCRs in triplex and single.

Dilution	TS Triplex	TS Single	GS rRT-PCR	TS Triplex	TS Single	GS rRT-PCR
		Serotype 1			Serotype 2	
-1	POS (18.0±0.1)	POS (16.6±0.1)	POS (21.8)	POS (18.9±0.1)	POS (15.7±0.1)	POS (18.9)
-2	POS (22.7±0.4)	POS (20.8±0.3)	POS (24.7±0.1)	POS (22.2±0.3)	POS (18.8±0.2)	POS (22.1±0.1)
-3	POS (26.3±0.3)	POS (24.7±0.2)	POS (27.1±1.5)	POS (25.8±0.6)	POS (22.4±0.2)	POS (25.4±0.5)
-4	POS (29.9±0.2)	POS (28.3±0.4)	POS (32.1±0.3)	POS (29.6±0.3)	POS (26.5±0.3)	POS (29.6±0.0)
-5	POS (33.8±0.5)	POS (32.9±0.6)	INC (35.6±0.6)	POS (33.4±0.5)	POS (29.9±0.3)	POS (33.0±0.4)
-6	INC (38.2±1.3)	INC (36.5±0.3)	NEG	INC (36.6±0.5)	POS (32.9±0.2)	INC (36.1±0.0)
-7	NEG	NEG	NEG	INC (39.4±1.0)	INC (36.2±0.4)	INC (38.8±1.8)
		Serotype 3			Serotype 4	
-1	POS (19.4±0.6)	POS (17.5±0.1)	POS (21.9)	POS (18.1±0.1)	POS (17.0±0.4)	POS (21.5)
-2	POS (23.7±0.4)	POS (21.0±0.2)	POS (25.4±0.4)	POS (21.3±0.1)	POS (20.0±0.2)	POS (24.4±0.1)
-3	POS (26.5±0.6)	POS (24.8±0.3)	POS (28.8±0.7)	POS (24.7±0.1)	POS (23.2±0.4)	POS (27.8±0.3)
-4	POS (29.9±0.9)	POS (26.8±0.2)	POS (32.8±0.1)	POS (28.6±0.3)	POS (26.9±0.2)	POS (31.9±0.0)
-5	POS (33.4±1.3)	POS (30.5±0.4)	INC (35.3±0.2)	POS (32.1±0.2)	POS (30.9±0.3)	INC (35.4±0.3)
-6	INC (38.8±2.5)	INC (37.8±2.7)	NEG	INC (35.4±0.2)	POS (34.4±0.5)	INC (38.9±1.6)
-7	NEG	INC (37.7±2.7)	NEG	INC (38.9±1.3)	INC (39.4±1.3)	NEG
		Serotype 5			Serotype 6	
-1	POS (17.2±0.1)	POS (14.0±0.1)	POS (16.2)	POS (19.7±0.1)	POS (14.3±0.1)	POS (18)
-2	POS (20.1±0.2)	POS (16.9±0.2)	POS (19.5±0.0)	POS (22.1±0.7)	POS (17.7±0.6)	POS (20.7±0.9)
-3	POS (23.5±0.1)	POS (20.1±0.1)	POS (22.6±0.4)	POS (25.4±0.7)	POS (20.8±0.4)	POS (23.3±0.8)
-4	POS (27.3±0.7)	POS (23.9±0.2)	POS (26.7±0.3)	POS (29.2±0.8)	POS (24.7±0.8)	POS (27.4±1.5)
-5	POS (31.2±0.3)	POS (27.3±0.5)	POS (32.5±3.0)	POS (33.4±0.9)	POS (28.9±0.7)	POS (31.7±0.9)
-6	POS (34.7±0.4)	POS (30.5±0.3)	POS (33.6±0.5)	POS (33.4±0.9)	POS (33.5±0.4)	INC (38.3±2.4)
-7	INC (38.2±1.1)	POS (34.1±0.6)	INC (37.0±0.2)	NEG	INC (36.5±1.7)	NEG
		Serotype 7			Serotype 8	
-1	POS (22.7±0.1)	POS (18.1±0.4)	POS (19.4)	not done	not done	not done
-2	POS (26.1±0.2)	POS (21.9±0.1)	POS (23.0±0.4)	POS (21.6±1.2)	POS (19.5±0.7)	POS (22.9±0.9)
-3	POS (29.4±0.2)	POS (25.2±0.4)	POS (26.9±0.1)	POS (25.1±0.7)	POS (23.4±1.4)	POS (26.6±0.6)
-4	POS (33.1±0.5)	POS (28.2±0.3)	POS (30.7±0.4)	POS (29.8±0.4)	POS (28.0±0.1)	POS (30.5±0.3)
-5	INC (37.6±1.1)	POS (32.1±0.3)	POS (33.6±0.6)	POS (34.1±0.8)	POS (31.9±0.3)	POS (34.3±0.3)
-6	INC (39.7±0.6)	INC (36.2±0.7)	INC (37.4±0.1)	INC (35.4±0.2)	INC (35.9±0.2)	INC (37.3±0.8)
-7	NEG	NEG	INC (38.8±1.8)	INC (39.6±0.8)	INC (39.5±1.0)	NEG
		Serotype 9				
-1	POS (17.4±0.1)	POS (15.5±0.1)	POS (17.4)			
-2	POS (20.4±0.1)	POS (17.7±0.1)	POS (21.6±0.1)			
-3	POS (23.7±0.2)	POS (21.4±0.7)	POS (25.2±0.5)			
-4	POS (27.3±0.1)	POS (24.6±0.7)	POS (28.2±0.4)			
-5	POS (31.2±0.4)	POS (28.7±0.8)	POS (32.2±0.5)			
-6	POS (34.1±0.4)	POS (32.3±0.8)	INC (35.4±0.3)			
-7	INC (37.7±0.6)	INC (35.2±0.6)	INC (39.0±1.4)			

POS: positive; INC: inconclusive; NEG: negative; (Ct value average ± standard deviation)

Table S2. Results obtained on field AHSV isolates in cell culture collection.

Strain (source)	Cell line	GS rRT-PCR	Triplex TS rRT-PCR			Triplex TS rRT-PCR			Triplex TS rRT-PCR		
			Ser 1	Ser 6	Ser 8	Ser 2	Ser 4	Ser 9	Ser 3	Ser 5	Ser 7
SEN2007 st2 (TPI)	B	POS (18.4)	Neg	Neg	Neg	POS (17.2)	Neg	Neg	Neg	Neg	Neg
SEN2007 st7 (TPI)	V	POS (18.1)	Neg	Neg	POS (32.8)†	Neg	Neg	Neg	Neg	Neg	POS (19.4)
KEN2006 st9 (TPI)	KC	POS (18.8)	Neg	Neg	Neg	Neg	Neg	POS (19.9)	Neg	Neg	Neg
KEN2007 st4 (TPI)	B	POS (21.0)	Neg	Neg	Neg	Neg	POS (18.7)	Neg	Neg	Neg	Neg
GHA2010 st2 (TPI)	V	POS (31.5)	Neg	Neg	Neg	POS (32.6)	Neg	Neg	Neg	Neg	Neg
ETH2010 st6 (TPI)	B	POS (19.1)	Neg	POS (17.4)	Neg	Neg	Neg	Neg	Neg	Neg	Neg
ETH2010 st2 (TPI)	V	POS (24.3)	Neg	Neg	INC (36.5)†	POS (17.2)	Neg	Neg	Neg	Neg	Neg
ETH2010 st4 (TPI)	B	POS (32.6)	Neg	Neg	Neg	Neg	POS (17.7)	Neg	Neg	Neg	Neg
ETH2010 st8 (TPI)	B	POS (23.6)	Neg	Neg	POS (18.8)	Neg	Neg	Neg	Neg	Neg	Neg
ETH2010 st9 (TPI)	B	POS (24.6)	Neg	Neg	Neg	Neg	Neg	POS (26.1)	Neg	Neg	Neg
KEN st1 (CVR)	V	POS (21.4)	POS (20.6)	Neg	Neg	Neg	Neg	Neg	Neg	Neg	Neg
KEN st4 (CVR)	B	POS (19.6)	Neg	Neg	Neg	Neg	POS (17.5)	Neg	Neg	Neg	Neg
KEN st5 (CVR)	B	POS (16.8)	Neg	Neg	Neg	Neg	Neg	Neg	Neg	POS (18.2)	Neg
KEN st7 (CVR)	B	POS (17.1)	Neg	Neg	Neg	Neg	Neg	Neg	Neg	Neg	POS (21.3)
KEN st8 (CVR)	B	POS (20.5)	Neg	Neg	POS (20.9)	Neg	Neg	Neg	Neg	Neg	Neg
KEN st9 (CVR)	B	POS (26.8)	Neg	Neg	Neg	Neg	Neg	POS (19.5)	Neg	Neg	Neg
KEN2015 st2 (CVR)*	B	POS (18.2)	Neg	Neg	Neg	POS (16.2	Neg	Neg	Neg	Neg	Neg
KEN2013 st4 (CVR)	B	POS (20.2)	Neg	Neg	Neg	Neg	POS (17.9)	Neg	Neg	Neg	Neg
KEN2015 st7 (CVR)*	B	POS (18.6)	Neg	Neg	Neg	Neg	Neg	Neg	Neg	Neg	POS (19.7)
KEN2016 st4 (CVR)*	B	POS (22.3)	Neg	Neg	Neg	Neg	POS (23.7)	Neg	Neg	Neg	Neg
KEN2017 st5 (CVR)*	V	POS (19.4)	Neg	Neg	INC (35.3)†	Neg	Neg	Neg	Neg	POS (18.5)	Neg
SPA1988 st4 (LCV)	V	POS (16.2)	Neg	Neg	POS (31.9)†	Neg	POS (14)	Neg	Neg	Neg	Neg
SPA1989 st4 (LCV)	V	POS (18.4)	Neg	Neg	INC (36.7)†	Neg	POS (15.4)	Neg	Neg	Neg	Neg
SPA1990 st4 (LCV)	V	POS (21.8)	Neg	Neg	POS (30.7)†	Neg	POS (18.4)	Neg	Neg	Neg	Neg
SPA1987 st3 (LCV)	V	POS (19.7)	Neg	Neg	POS (32.8)†	Neg	Neg	Neg	POS (15.5)	Neg	Neg
SPA1987 st2 (LCV)	V	POS (19.4)	Neg	Neg	POS (33.1)†	POS (18.1)	Neg	Neg	Neg	Neg	Neg
THA2020 st1 (NIAH)*	V	POS (31.7)	POS (24.7)	Neg	Neg	Neg	Neg	Neg	Neg	Neg	Neg

TPI: The Pirbright Institute; CVR: Central Veterinary Research Laboratory; LCV: Laboratorio Central de Veterinaria; NIAH: National Institute of Animal Health; B: BHK; V: Vero
Neg: negative; POS: positive (Ct value); INC: inconclusive (Ct value)
*Virus isolated in the LCV from clinical samples received from this source
†Negative in single TS rRT-PCR serotype 8

Table S3. Results obtained on clinical samples (16 spleen and 3 brain) from Spanish outbreak during the period 1987 – 1988.

Identification	Year	GS rRT-PCR	Triplex TS rRT-PCR			Triplex TS rRT-PCR			Triplex TS rRT-PCR		
			Ser 1	Ser 6	Ser 8	Ser 2	Ser 4	Ser 9	Ser 3	Ser 5	Ser 7
1528/87 (1)	1987	POS (25.7)	Neg	Neg	Neg	Neg	POS (22.8)	Neg	Neg	Neg	Neg
1528/87 (2)	1987	POS (26.6)	Neg	Neg	Neg	Neg	POS (21.6)	Neg	Neg	Neg	Neg
1537/87	1987	POS (25.1)	Neg	Neg	Neg	Neg	POS (21.4)	Neg	INC (36.01)	Neg	Neg
1560/87	1987	POS (24.8)	Neg	Neg	Neg	Neg	POS (19.4)	Neg	Neg	Neg	Neg
1661/87	1987	POS (21.8)	Neg	Neg	Neg	Neg	POS (18.8)	Neg	Neg	Neg	Neg
1694/87 (28)	1987	POS (21.6)	Neg	Neg	Neg	Neg	POS (19.0)	POS (27.7)	POS (26.6)	Neg	Neg
1724/87	1987	POS (22.1)	Neg	Neg	Neg	Neg	POS (19.8)	Neg	Neg	Neg	Neg
1735/87	1987	POS (22.4)	Neg	Neg	Neg	Neg	POS (18.9)	POS (27.2)	POS (32.1)	Neg	Neg
1775/87*	1987	POS (28.1)	Neg	Neg	Neg	Neg	INC (36.0)	Neg	POS (25.4)	Neg	Neg
1841/87*	1987	POS (30.6)	Neg	Neg	Neg	Neg	INC (35.6)	Neg	POS (29.8)	Neg	Neg
1841/87 (1)	1987	POS (32.3)	Neg	Neg	Neg	Neg	POS (30.2)	Neg	POS (34.2)	Neg	Neg

1903/87*	1987	POS (30.6)	Neg	Neg	Neg	POS (31.4)	POS (32.9)	Neg	Neg	Neg	Neg
1955/87	1987	POS (29.1)	Neg	Neg	Neg	Neg	POS (28.9)	POS (31.7)	POS (34.4)	POS (33.6)	Neg
1722/88 (3)	1988	POS (22.8)	Neg	Neg	Neg	Neg	POS (21.5)	Neg	POS (34.9)	Neg	Neg
1722/88 (5)	1988	POS (24.6)	Neg	Neg	Neg	Neg	POS (21.8)	Neg	Neg	Neg	Neg
1722/88 (12)	1988	POS (22.4)	Neg	Neg	Neg	Neg	POS (20.0)	Neg	Neg	Neg	Neg
1772/88 (7)	1988	POS (23.3)	Neg	Neg	Neg	Neg	POS (20.6)	Neg	Neg	Neg	Neg
1832/88 (3)	1988	POS (25.2)	Neg	Neg	Neg	Neg	POS (23.0)	Neg	POS (29.4)	Neg	Neg
1943/88 (4)	1988	POS (25.2)	Neg	Neg	Neg	Neg	POS (23.6)	Neg	POS (28.7)	Neg	Neg

Neg: negative; POS: positive (Ct value); INC: inconclusive (Ct value)

*Brain samples. Remainder samples were spleen.

Table S4. Results obtained on clinical samples from AHS outbreak in Thailand (2020). Source of samples: National Institute of Animal Health, Thailand.

Identification	Year	GS rRT-PCR	Triplex TS rRT-PCR			Triplex TS rRT-PCR			Triplex TS rRT-PCR		
			Ser 1	Ser 6	Ser 8	Ser 2	Ser 4	Ser 9	Ser 3	Ser 5	Ser 7
R.1127/20 (20)	EDTA Blood	POS (22.2)	POS (26.4)	Neg	Neg	Neg	Neg	Neg	Neg	Neg	Neg
R.1127/20 (21)	EDTA Blood	POS (22.2)	POS (26.1)	Neg	Neg	Neg	Neg	Neg	Neg	Neg	Neg
R.1127/20 (22)	EDTA Blood	POS (21.7)	POS (24.6)	Neg	Neg	Neg	Neg	Neg	Neg	Neg	Neg
R.1127/20 (23)	EDTA Blood	POS (22.8)	POS (25.0)	Neg	Neg	Neg	Neg	Neg	Neg	Neg	Neg
R.1127/20 (24)	EDTA Blood	POS (24.7)	POS (28.1)	Neg	Neg	Neg	Neg	Neg	Neg	Neg	Neg
R.1127/20 (25)	EDTA Blood	POS (28.1)	POS (33.5)	Neg	Neg	Neg	Neg	Neg	Neg	Neg	Neg
R.1127/20 (26)	EDTA Blood	POS (21.4)	POS (30.3)	Neg	Neg	Neg	Neg	Neg	Neg	Neg	Neg
R.1127/20 (27)	Tissue	POS (23.8)	POS (28.5)	Neg	Neg	Neg	Neg	Neg	Neg	Neg	Neg
R.1127/20 (28)	Tissue	POS (22.1)	POS (25.4)	Neg	Neg	Neg	Neg	Neg	Neg	Neg	Neg
R.1127/20 (29)	Tissue	POS (24.3)	POS (28.1)	Neg	Neg	Neg	Neg	Neg	Neg	Neg	Neg
R.1127/20 (30)	RNA	POS (25.3)	POS (24.4)	Neg	Neg	Neg	Neg	Neg	Neg	Neg	Neg
R.1127/20 (31)	RNA	POS (24.5)	POS (30.7)	Neg	Neg	Neg	Neg	Neg	Neg	Neg	Neg
R.1127/20 (32)	RNA	POS (24.5)	POS (27.9)	Neg	Neg	Neg	Neg	Neg	Neg	Neg	Neg
R.1127/20 (33)	RNA	POS (18.2)	POS (21.3)	Neg	Neg	Neg	Neg	Neg	POS (33.4)	Neg	Neg
R.1127/20 (35)	RNA	POS (25.6)	POS (32.2)	Neg	Neg	Neg	Neg	Neg	Neg	Neg	Neg

Neg: negative; POS: positive (Ct value)

Table S5. Results obtained on clinical samples from AHS outbreak in Nigeria (2022-23). Source of samples: National Veterinary Research Institute, Nigeria.

Identification	Year	GS rRT-PCR	Triplex TS rRT-PCR			Triplex TS rRT-PCR			Triplex TS rRT-PCR		
			Ser 1	Ser 6	Ser 8	Ser 2	Ser 4	Ser 9	Ser 3	Ser 5	Ser 7
R.1507/23 (8)	Tissue (spleen)	POS (22.8)	Neg	Neg	Neg	Neg	Neg	Neg	Neg	Neg	POS (23.2)
R.1507/23 (9)	Tissue (liver)	POS (24.5)	Neg	Neg	Neg	Neg	Neg	Neg	Neg	Neg	POS (24.2)
R.1507/23 (10)	Tissue (heart)	POS (21.9)	Neg	Neg	Neg	Neg	Neg	Neg	Neg	Neg	POS (22.4)
R.1507/23 (11)	Tissue (trachea)	POS (30.9)	Neg	Neg	Neg	Neg	Neg	Neg	Neg	Neg	POS (31.7)
R.1507/23 (12)	Tissue (viscera)	POS (23.6)	Neg	Neg	Neg	Neg	Neg	Neg	Neg	Neg	POS (23.8)
R.1507/23 (13)	Tissue (viscera)	POS (26.3)	Neg	Neg	Neg	Neg	Neg	Neg	Neg	Neg	POS (26.6)
R.1507/23 (14)	Tissue (trachea)	POS (29.5)	Neg	Neg	Neg	Neg	Neg	Neg	Neg	Neg	POS (29.7)
R.1507/23 (15)	Tissue (heart)	POS (24.6)	Neg	Neg	Neg	Neg	Neg	Neg	Neg	Neg	POS (24.7)
R.1507/23 (16)	Tissue (liver)	POS (24.9)	Neg	Neg	Neg	Neg	Neg	Neg	Neg	Neg	POS (25.0)
R.1507/23 (17)	Tissue (spleen)	POS (20.8)	Neg	Neg	Neg	Neg	Neg	Neg	Neg	Neg	POS (21.9)

Neg: negative; POS: positive (Ct value)

Table S6. Results obtained on untyped clinical samples from Kenia (2015 - 2017). Source of samples: Central Veterinary Research Laboratory, Dubai, UAE

Identification	Type	GS rRT-PCR	Triplex TS rRT-PCR Ser 1	Ser 6	Ser 8	Triplex TS rRT-PCR Ser 2	Ser 4	Ser 9	Triplex TS rRT-PCR Ser 3	Ser 5	Ser 7
2441/15 17	B	POS (30.6)	Neg	Neg	Neg	Neg	Neg	POS (27.7)	Neg	Neg	Neg
2441/15 19	B	POS (26.9)	Neg	Neg	Neg	POS (25.9)	Neg	Neg	Neg	Neg	Neg
2441/15 20	B	POS (31.3)	POS (31.4)	Neg	Neg	Neg	Neg	Neg	Neg	Neg	Neg
2441/15 13	B	POS (24.8)	Neg	Neg	Neg	Neg	Neg	Neg	Neg	POS (23.9)	Neg
2782/15 1	B	POS (29.8)	Neg	Neg	Neg	POS (27.3)	Neg	Neg	Neg	Neg	Neg
2782/15 2	B	POS (32.5)	Neg	Neg	Neg	POS (31.6)	Neg	Neg	Neg	Neg	Neg
2782/15 3	B	POS (24.9)	Neg	Neg	Neg	POS (23.6)	Neg	Neg	Neg	Neg	Neg
256/16 2	B	POS (26.9)	Neg	Neg	Neg	Neg	POS (23.0)	Neg	Neg	Neg	Neg
256/16 3	B	POS (27.4)	Neg	Neg	Neg	Neg	Neg	POS (23.7)	Neg	Neg	Neg
256/16 4	B	POS (27.2)	Neg	Neg	Neg	Neg	Neg	Neg	Neg	Neg	POS (26.5)
377/16 2	B	POS (29.8)	Neg	Neg	Neg	Neg	POS (27.4)	Neg	Neg	Neg	Neg
377/16 3	B	POS (26.3)	Neg	Neg	Neg	Neg	POS (23.4)	Neg	Neg	Neg	Neg
841/16 8	B	POS (28.0)	Neg	Neg	Neg	Neg	POS (24.8)	Neg	Neg	Neg	Neg
841/16 9	B	POS (25.8)	Neg	Neg	Neg	Neg	Neg	Neg	Neg	Neg	POS (25.9)
841/16 10	B	POS (28.1)	Neg	Neg	Neg	POS (22.8)	Neg	Neg	Neg	Neg	Neg
1766/16 2	B	POS (30.3)	Neg	Neg	Neg	POS (28.7)	Neg	Neg	Neg	Neg	Neg
1766/16 3	B	POS (29.8)	Neg	Neg	Neg	POS (27.0)	Neg	Neg	Neg	Neg	Neg
1766/16 4	B	POS (29.1)	Neg	Neg	Neg	POS (27.1)	Neg	Neg	Neg	Neg	Neg
1766/16 5	B	POS (29.5)	Neg	Neg	Neg	Neg	POS (26.2)	Neg	Neg	Neg	Neg
2459/16 2	B	POS (31.6)	Neg	Neg	Neg	Neg	POS (26.8)	Neg	Neg	Neg	Neg
2459/16 3	B	POS (30.0)	Neg	Neg	Neg	Neg	Neg	POS (27.5)	Neg	Neg	Neg
2459/16 4	B	POS (28.0)	Neg	Neg	Neg	Neg	POS (23.9)	Neg	Neg	Neg	Neg
2459/16 5	B	POS (30.6)	Neg	Neg	Neg	Neg	Neg	Neg	POS (24.7)	Neg	Neg
2772/16 1	B	POS (25.1)	Neg	Neg	Neg	Neg	Neg	POS (23.1)	Neg	Neg	Neg
1083/17 1	B	POS (23.9)	Neg	Neg	Neg	Neg	Neg	Neg	Neg	POS (25.7)	Neg
1838/17 1	B	POS (29.5)	Neg	Neg	Neg	Neg	Neg	Neg	Neg	POS (28.9)	Neg
2441/15 11	S	POS (25.3)	Neg	Neg	Neg	Neg	Neg	Neg	Neg	POS (26.0)	Neg
2441/15 16	S	POS (28.7)	Neg	Neg	Neg	Neg	POS (26.2)	Neg	Neg	Neg	Neg
2782/15 4	LI	POS (28.9)	Neg	Neg	Neg	POS (28.0)	Neg	Neg	Neg	Neg	Neg
2782/15 5	S	POS (27.3)	Neg	Neg	Neg	POS (25.2)	Neg	Neg	Neg	Neg	Neg
2782/15 6	LU	POS (20.0)	Neg	Neg	Neg	POS (17.2)	Neg	Neg	Neg	Neg	Neg
2782/15 7	H	POS (32.3)	Neg	Neg	Neg	POS (32.6)	Neg	Neg	Neg	Neg	Neg
256/16 5	LU	POS (33.3)	Neg	Neg	Neg	Neg	POS (30.9)	Neg	Neg	Neg	Neg
256/16 6	H	POS (32.5)	Neg	Neg	Neg	Neg	POS (19.4)	Neg	Neg	Neg	Neg
256/16 7	S	POS (24.7)	Neg	Neg	Neg	Neg	POS (22.1)	Neg	Neg	Neg	Neg
256/16 8	LI	POS (25.6)	Neg	Neg	Neg	Neg	POS (23.1)	Neg	Neg	Neg	Neg
256/16 9	LU	POS (31.5)	Neg	Neg	Neg	Neg	Neg	POS (28.6)	Neg	Neg	Neg
256/16 10	H	POS (22.4)	Neg	Neg	Neg	Neg	Neg	POS (19.3)	Neg	Neg	Neg
256/16 11	S	POS (27.1)	Neg	Neg	Neg	Neg	Neg	POS (24.5)	Neg	Neg	Neg
256/16 12	LI	POS (26.3)	Neg	Neg	Neg	Neg	Neg	POS (22.9)	Neg	Neg	Neg
1766/16 8	H	POS (29.4)	Neg	Neg	Neg	Neg	POS (25.6)	Neg	Neg	Neg	Neg
1766/16 9	LU	POS (32.4)	Neg	Neg	Neg	Neg	POS (28.9)	Neg	Neg	Neg	Neg
2459/16 6	LU	POS (24.4)	Neg	Neg	Neg	Neg	Neg	Neg	Neg	Neg	POS (23.6)
2459/16 9	LI	POS (26.4)	Neg	Neg	Neg	Neg	Neg	Neg	Neg	Neg	POS (25.4)
2772/16 3	H	POS (26.8)	Neg	Neg	Neg	Neg	POS (24.1)	Neg	Neg	Neg	Neg
2772/16 4	S	POS (26.9)	Neg	Neg	Neg	Neg	POS (25.2)	Neg	Neg	Neg	Neg
2772/16 5	S	POS (26.5)	Neg	Neg	Neg	Neg	POS (24.7)	Neg	Neg	Neg	Neg

1083/17 3	LU	POS (21.5)	Neg	Neg	Neg	Neg	Neg	Neg	Neg	POS (22.6)	Neg
1083/17 5	S	POS (23.7)	Neg	Neg	Neg	Neg	Neg	Neg	Neg	POS (24.6)	Neg
1083/17 7	LU	POS (17.4)	Neg	Neg	Neg	Neg	Neg	Neg	Neg	POS (18.8)	Neg
1083/17 8	H	POS (23.2)	Neg	Neg	Neg	Neg	Neg	Neg	Neg	POS (28.2)	Neg
1083/17 9	LI	POS (19.5)	Neg	Neg	Neg	Neg	Neg	Neg	Neg	POS (20.5)	Neg

Neg: negative; POS: positive (Ct value)
B: EDTA blood; S: spleen; LU: lung; LI: liver; H: heart

Table S7. Results obtained on untyped clinical samples from WOAH Ring Trial (2015). Source of samples: Onderstepoort Veterinary Institute, South-Africa.

Sample	GS rRT-PCR	Triplex TS rRT-PCR			Triplex TS rRT-PCR			Triplex TS rRT-PCR		
		**Ser 1**	**Ser 6**	**Ser 8**	**Ser 2**	**Ser 4**	**Ser 9**	**Ser 3**	**Ser 5**	**Ser 7**
1	INC (36.4)	Neg	Neg	Neg	Neg	Neg	Neg	POS (34.9)	Neg	Neg
2	POS (34.8)	Neg	Neg	Neg	Neg	Neg	Neg	POS (32.5)	Neg	Neg
3	POS (31.1)	Neg	Neg	Neg	Neg	Neg	Neg	Neg	Neg	Neg
4	POS (27.4)	Neg	Neg	Neg	Neg	POS (27.0)	Neg	Neg	Neg	Neg
5	POS (24.7)	Neg	Neg	Neg	Neg	Neg	POS (24.6)	Neg	Neg	Neg
6	POS (29.0)	Neg	INC (37.0)	Neg	Neg	Neg	Neg	Neg	Neg	Neg
7	POS (25.7)	Neg	Neg	Neg	Neg	POS (24.8)	Neg	Neg	Neg	Neg
8	Neg	Neg	Neg	Neg	Neg	Neg	Neg	Neg	Neg	Neg
9	POS (32.4)	Neg	Neg	Neg	Neg	Neg	Neg	Neg	Neg	POS (30.8)
10	POS (31.5)	Neg	Neg	Neg	Neg	Neg	POS (31.2)	Neg	Neg	Neg
11	POS (28.3)	Neg	Neg	Neg	Neg	Neg	Neg	Neg	Neg	POS (30.6)
12	POS (31.7)	Neg	POS (30.9)	Neg	Neg	Neg	Neg	Neg	Neg	Neg
13	Neg	Neg	Neg	Neg	Neg	Neg	Neg	Neg	Neg	Neg
14	Neg	Neg	Neg	Neg	Neg	Neg	Neg	Neg	Neg	Neg
15	POS (26.8)	Neg	Neg	Neg	INC (36.7)	Neg	Neg	Neg	Neg	Neg
16	POS (26.6)	Neg	Neg	Neg	INC (36.2)	Neg	Neg	Neg	Neg	Neg
17	POS (24.2)	Neg	Neg	Neg	Neg	POS (23.6)	Neg	Neg	Neg	Neg
18	POS (26.8)	Neg	Neg	Neg	INC (37.3)	Neg	Neg	Neg	Neg	Neg
19	POS (28.5)	Neg	Neg	Neg	Neg	Neg	POS (28.7)	Neg	Neg	Neg
20	POS (32.7)	Neg	Neg	Neg	Neg	Neg	Neg	Neg	Neg	POS (33.9)
21	POS (26.9)	Neg	Neg	Neg	POS (34.8)	Neg	Neg	Neg	Neg	Neg
22	POS (30.2)	Neg	Neg	Neg	Neg	Neg	Neg	Neg	Neg	POS (29.0)
23	POS (27.8)	Neg	Neg	Neg	Neg	Neg	POS (27.4)	Neg	Neg	Neg
24	POS (28.5)	Neg	Neg	Neg	Neg	Neg	POS (28.0)	Neg	Neg	Neg
25	POS (26.8)	Neg	Neg	Neg	POS (31.9)	Neg	Neg	Neg	Neg	Neg
26	POS (29.0)	POS (34.9)	Neg	Neg	Neg	Neg	Neg	Neg	Neg	Neg
27	POS (28.1)	Neg	Neg	Neg	Neg	POS (26.6)	Neg	Neg	Neg	Neg
28	POS (30.9)	Neg	Neg	Neg	Neg	Neg	Neg	Neg	Neg	POS (30.3)
29	POS (28.7)	Neg	Neg	Neg	Neg	Neg	POS (30.7)	Neg	Neg	Neg
30	POS (28.4)	Neg	Neg	Neg	INC (35.6)	Neg	Neg	Neg	Neg	Neg
31	Neg	Neg	Neg	Neg	Neg	Neg	Neg	Neg	Neg	Neg
32	POS (25.9)	Neg	Neg	Neg	Neg	Neg	POS (26.3)	Neg	Neg	Neg
33	POS (27.7)	Neg	Neg	Neg	Neg	Neg	POS (26.3)	Neg	Neg	Neg
34	POS (31.9)	Neg	Neg	Neg	Neg	Neg	POS (33.9)	Neg	Neg	Neg
35	POS (27.8)	Neg	Neg	Neg	Neg	Neg	Neg	Neg	Neg	POS (30.6)
36	POS (22.6)	Neg	Neg	Neg	Neg	Neg	POS (27.0)	Neg	Neg	Neg
37	POS (28.6)	Neg	Neg	Neg	Neg	Neg	POS (33.3)	Neg	Neg	Neg
38	Neg	Neg	Neg	Neg	Neg	Neg	Neg	Neg	Neg	Neg

39	POS (30.6)	Neg	Neg	Neg	Neg	INC (36.6)	Neg	Neg	Neg	Neg
40	POS (26.1)	Neg	Neg	Neg	POS (33.5)	Neg	Neg	Neg	Neg	Neg
41	POS (26.7)	Neg	Neg	Neg	Neg	Neg	POS (26.3)	Neg	Neg	Neg
42	Neg	Neg	Neg	Neg	Neg	Neg	Neg	Neg	Neg	Neg
43	POS (28.6)	Neg	Neg	Neg	Neg	Neg	Neg	POS (32.2)	Neg	Neg
44	POS (28.9)	Neg	Neg	Neg	Neg	POS (32.5)	Neg	Neg	Neg	Neg
45	Neg	Neg	Neg	Neg	Neg	Neg	Neg	Neg	Neg	Neg
46	Neg	Neg	Neg	Neg	Neg	Neg	Neg	Neg	Neg	Neg
47	Neg	Neg	Neg	Neg	Neg	Neg	Neg	Neg	Neg	Neg
48	Neg	Neg	Neg	Neg	Neg	Neg	Neg	Neg	Neg	Neg
49	Neg	Neg	Neg	Neg	Neg	Neg	Neg	Neg	Neg	Neg
50	POS (26.7)	Neg	Neg	Neg	POS (32.4)	Neg	Neg	Neg	Neg	Neg

Neg: negative; POS: positive (Ct value); INC: inconclusive (Ct value)
